# COMPLEX FRACTURES OF THE DISTAL RADIUS: ANALYSIS OF OSTEOSYNTHESIS USING SPANNING PLATES

**DOI:** 10.1590/1413-785220243201e278038

**Published:** 2025-04-07

**Authors:** TÚLIO FELÍCIO DA CUNHA RODRIGUES, AFRÂNIO DONATO DE FREITAS, JOÃO ANTONIO CÔRTES VIEIRA, JUAN CAMILO ORTEGA RIVERA, RODRIGO MITRE COTTA

**Affiliations:** 1 Fundação Hospitalar São Francisco de Assis, Belo Horizonte, MG, Brazil.

**Keywords:** Analysis, Internal Fracture Fixation, Radius Fractures, Bone Plates, Análise, Fixação Interna de Fraturas, Fraturas do Rádio, Placas Ósseas

## Abstract

**Objective:**

To radiographically evaluate the postoperative results of AO 2R3C3 type distal radius fractures treated using the spanning plate technique, associated or not with other fixation methods.

**Methods:**

Retrospective observational study that evaluated 14 patients, aged 29 to 80 years, who underwent osteosynthesis, from November 2021 to June 2022. For radiographic measurement, the following parameters were defined: ulnar variance, volar inclination, radial inclination and step articulate.

**Results:**

The mean value of radial inclination was 13.70 degrees, volar inclination was 0.85 degrees, ulnar variance was 0.60mm and joint step was 0.78mm.

**Conclusion:**

Fixation using spanning plate is an excellent alternative in the management of complex fractures of the distal radius. ***Level of Evidence IV, Case Series.***

## RESUMO

**Objetivo:**

Avaliar radiograficamente os resultados pós-operatórios de fraturas do rádio distal do tipo AO 2R3C3 tratados pela técnica de placa distração associada ou não a outros métodos de fixação.

**Métodos:**

Estudo observacional retrospectivo que avaliou 14 pacientes, de 29 a 80 anos, submetidos à osteossíntese, no período de novembro de 2021 a junho de 2022. Para mensuração radiográfica, foram definidos como parâmetros: variância ulnar, inclinação volar, inclinação radial e degrau articular.

**Resultados:**

O valor médio da inclinação radial foi de 13,70 graus, da inclinação volar foi de 0,85 graus, da variância ulnar de 0,60mm e do degrau articular foi de 0,78mm.

**Conclusão:**

A fixação com o uso de placas de distração dorsal é uma excelente alternativa no manejo de fraturas complexas do rádio distal. **Nível de evidência IV, Série de Casos.**

## INTRODUCTION

Distal radius fractures are serious injuries with an unclear prognosis, regardless of the treatment applied, and the presence of associated injuries makes the outcome even more unpredictable. Although distal third radius fractures are among the most frequent in orthopedic emergency care, being the most common fracture of the upper limb, treating them remains a challenge even with many fixation methods available, and the search for more appropriate solutions is constant.

In the case of complex fractures, this challenge is even greater. It affects adults of varying ages, younger ones generally being victims of high-energy traumas, while older ones have fractures even in low kinetic energy accidents. Given their regularity, difficulty of treatment, wide age range affected, complications reflecting on work capacity and high social/economic cost, these fractures are reasons to constantly search for effective solutions.

**Figure 1:**
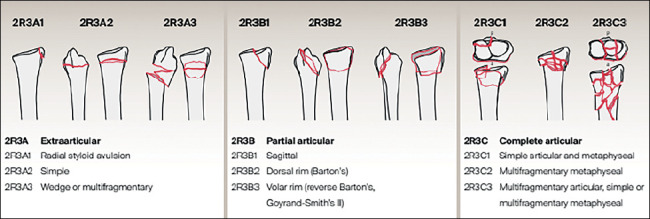
AO classification for distal radius fractures.

Source: AO/OTA Fracture and Dislocation Classification Long-bone Fractures - Poster.

Most fractures are extra-articular, classified, according to the AO group, as type A. There are also the so-called partial joint fractures (AO type B) and complete joint fractures (AO type C).^2^ About 4.5% of complete joint fractures are complex, represented by the AO 2R3C3 subtype^3^ ^)^ (Figure 1). Complex articular distal radius fractures will be the object of this study.

There is a variety of proposed treatments in literature, with no consensus around obtained results. ^(^ ^4^ The goal is to restore the normal anatomy of the distal radius as precisely as possible, offering sufficient stability to allow wrist consolidation and maintenance of physiological biomechanics. ^(^ ^3^ ^)-(^ ^5^

Fractures that show instability do not present good results with conservative treatment. Therefore, surgical treatment should be favored and selected individually according to the fractures’ particularities. ^(^ ^5^ ^)-(^ ^7^

Complex joint fractures with multiple fragments are usually treated with internal fixation, using locked plates or external fixators with or without Kirschner wires. Locked plates offer great stability, but, due to their low availability, are not a solution for all cases. External fixators show satisfactory results but show a considerable number of infections in the pin path, loss of reduction, stiff fingers and discomfort with the material. ^(^ ^3^ ^),(^ ^6^ ^),(^ ^7^ ^),(^ ^9^

Spanning plates were first described by Burke and Singer in 1998, representing a method of relative stability, in which indirect reduction of the fracture is performed by ligamentotaxis and dorsal support, with a lower risk of infection and greater material tolerance, also allowing early partial weight support. ^(^ ^6^ ^)-(^ ^9^

The objective of this study is to radiographically evaluate the use of spanning plates, associated or not with other fixation methods, as a viable alternative to external fixators when treating complex articular distal radius fractures, in departments where locked plates are unavailable.

## MATERIALS AND METHODS

We performed a retrospective observational study at the São Francisco de Assis Hospital, in Belo Horizonte, Minas Gerais, Brazil, based on medical records and pre and postoperative radiographs of patients admitted to the Hand Surgery department who underwent surgery for complex distal radius fractures, between November 2021 and June 2022, using a spanning plate, associated or not with other synthesis materials.

The following exclusion criteria were used: patients with an immature skeleton, patients who did not undergo the proposed postoperative follow-up, patients with fractures of the ipsilateral upper limb (shoulder or elbow) and patients who had open fractures with extensive associated soft tissue injuries.

The patients’ basic sociodemographic data were obtained and recorded, such as: age, gender, mechanism and date of trauma, date of surgery, affected side and dominant hand.

Fractures were categorized before surgery, based on radiographs in anteroposterior (AP) and lateral views, according to the AO classification. There was a total of 14 fractures, all of them being type C3. In addition to instability criteria originally described by La Fontaine et al., ^(^ ^5^ new parameters from recent studies were used to prescribe surgery, such as: dorsal angulation greater than 20 degrees, dorsal comminution, shortening of the radius greater than 9 mm, involvement of the radiocarpal joint and the distal radioulnar joint, associated fracture of the ulna, deviation greater than 2 mm and age greater than 60 years old. ^(^ ^1^ ^),(^ ^2^

All surgeries were performed in the department by the same team of hand surgery specialists. Postoperative follow-up consisted of periodic consultations in the first, second, fourth, and eighth postoperative weeks, when plaque removal was scheduled.

**Figure 2:**
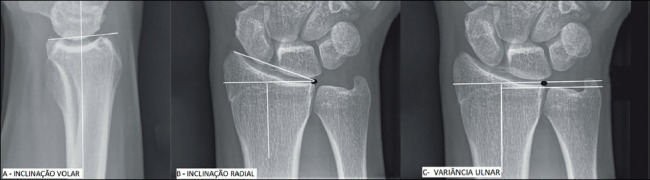
Measurements of radiographic parameters of the distal radius. A - Volar tilt. B - Radial inclination. C - Ulnar variance^2^.

In the eighth postoperative week, AP and lateral radiographs were used to measure ulnar variance, radial inclination, and volar tilt (Figure 2), as well as the articular step. Measurements were taken by two of the four authors, and the average was, then, recorded. The postoperative outcome was evaluated according to radiographic parameters of reduction present in literature. ^(^ ^10^

## SURGICAL TECHNIQUE

The patient is positioned in the horizontal dorsal decubitus position, under brachial plexus block. A radiolucent auxiliary table is used to support the limb, and a pneumatic arm tourniquet is placed on it. After asepsis and antisepsis of the limb, the tourniquet is inflated 100 mmHg above the patient’s systolic pressure.

**Figure 3:**
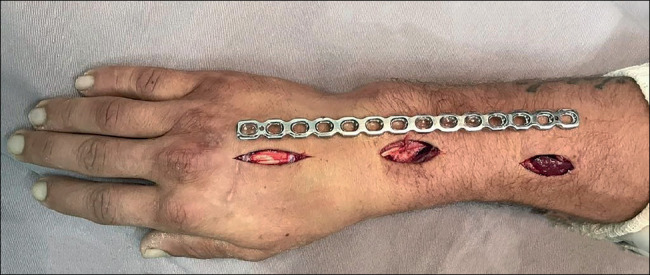
Surgical accesses: distal, intermediate, and proximal.

Source: Authors’ personal archive, 2021

**Figure 4:**
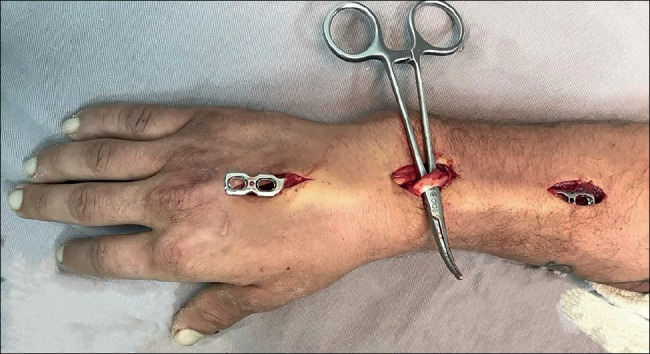
Release and clearance of the extensor pollicis longus (ELP) in the intermediate access.

Source: Authors’ personal archive, 2021.

**Figure 5:**
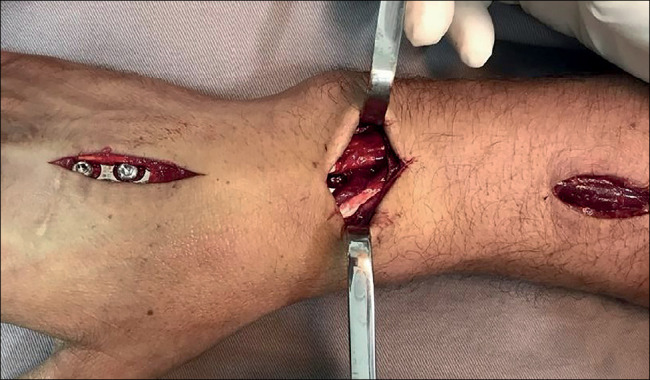
Final aspect of surgical wounds after fixation with the plate, the ELP tendon free.

Source: Authors’ personal archive, 2021.

A 12-hole 3.5 mm acetabular reconstruction plate is selected and placed directly on the back of the wrist. Fluoroscopy is used to identify the proximal and distal incision sites. The first incision is made on the dorsal aspect of the third metacarpal, approximately 4 cm long, dissecting until the periosteum is exposed, carefully removing the extensor tendon and other structures. A second longitudinal incision is made on the dorsal aspect of the distal metaphysis of the radius, of about 4 cm, guided by the plate’s length and positioning. The radial extensor tendons are identified, and blunt dissection is performed in the interval between them and the periosteum of the radius^6^ ^),(^ ^8^ ^),(^ ^11^ ^),(^ ^12^ ^)^ (Figure 3). A third, intermediate incision of 3 cm, slightly ulnar to Lister’s tubercle, is made to open the third extensor compartment and subsequently remove the long extensor tendon of the thumb (Figure 4). The extensor tendons in the fourth compartment are then elevated ulnarly, allowing the plate to be placed on the floor of that compartment. The plate is then carefully passed from distal to proximal, ensuring there is no extensor tendon clamping^6^ ^),(^ ^7^ ^),(^ ^9^ ^),(^ ^13^ ^)^ (Figure 5).

**Figure 6:**
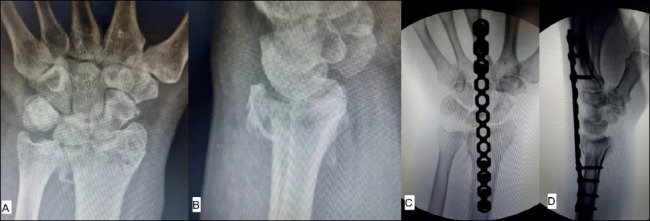
Preoperative radiographic images (A and B). Intraoperative fluoroscopic images after reduction and fixation (C and D).

Source: Authors’ personal archive, 2021.

The most distal screw of the metacarpal is then passed. The hand and forearm are kept in a neutral position, performing traction through the third finger. After traction, the proximal screw of the radius is fixated. Next, fixation is complemented with at least three bicortical screws in the third metacarpal and three bicortical screws in the radius. After fluoroscopy evaluation, the need for additional fixation with Kirschner wires or volar plate of small fragments is verified (Figure 6).

**Figure 7:**
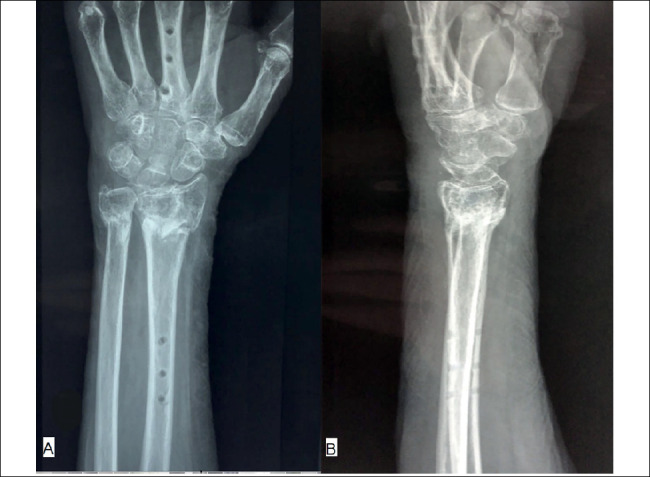
Radiographic images in AP (A) and lateral (B), two months postoperatively, after removing the spanning plate.

Source: Authors’ personal archive, 2021.

Immediately after surgery, a forearm plaster splint is applied in case Kirschner wires were used and is maintained until they are removed. In the absence of adjuvant fixation materials, immobilization is not used. The patient is stimulated and released to move their fingers, forearm and elbow extensively to avoid tendon adhesions and stiffness. Percutaneous pins, when used, are removed after six weeks. The plate is removed after eight weeks and, simultaneously with the surgery, the joint is manipulated to reestablish motion range and break possible fibrosis and adhesions. Physical therapy rehabilitation begins immediately after the synthesis is removed (Figure 7).

Possible complications include superficial and deep infections, loosening of the extensor mechanism (especially the third finger), irritation or even rupture of the extensor tendons, pseudoarthrosis, loosening of the synthesis material (plates and screws), loss of reduction. Complication rate in literature is low. ^(^ ^2^

## RESULTS

The study group consisted of 14 patients, five women (35.7%) and nine men (64.3%), with a mean age of 60.8 years, ranging from 29 to 80. Twelve of these were right-handed (85.7%) and two were left-handed (14.3%). Considering the total of fractures, thirteen were on the left wrist (92.85%) and only one was on the right wrist (7.15%). The most common injury mechanism was falls from one’s own height, eight patients (57.15%), and the other six (42.85%) were victims of traffic accidents.

All fractures were consolidated during the study. Radiographic results were obtained by measuring the angles of volar tilt and radial inclination, ulnar variance and articular step. For the measurement, wrist radiographs in AP and lateral radiographs were used, in the eighth postoperative week. Measurements were taken by two of the four authors, and the average among them was registered. Radial inclination had a mean of 13.70 º, ranging from 5 º to 22 º, representing a variance of 20.06 and a standard deviation of 4.48. Volar tilt had a mean of 0.85 º, ranging from 10 º to -3 º (3 degrees of dorsal tilt), configuring a variance of 8.26 and a standard deviation of 2.87. Ulnar variance had a mean of 0.60 mm, ranging from -1 mm to 4 mm, indicating a variance of 2.11 and a standard deviation of 1.45. Articular step had a mean of 0.78 mm, ranging from 0 to 3 mm, showing a variance of 1.16 and a standard deviation of 1.081. Table 1 shows the results.

**Table 1:** Recording of the averages obtained from radiographic parameters of each patient.

PATIENT	AGE	VOLAR TILT (DEGREES)	RADIAL INCLINATION (DEGREES)	ULNAR VARIANCE (MM)	ARTICULAR STEP (MM)
1	76	1	14	0	0
2	67	0	10	1	1
3	54	0	10	1	0
4	64	3	18	0	1
5	64	1	10	-1	2
6	63	1	10	0,5	0
7	58	0	5	0,5	0
8	54	0	15	3	3
9	51	0	18	0	3
10	49	-2	12	4	0
11	29	10	14	-1	0
12	73	-3	22	2	0
13	69	1	20	-1	1
14	80	0	14	-0,5	0

Source: Prepared by the authors.

**Table 2:** Acceptable radiographic parameters after fracture reduction.

PARAMETER	MEASURE
**DORSAL TILT**	**UP TO 10 º**
**RADIAL INCLINATION**	**GREATER THAN OR EQUAL TO 10 º**
**ULNAR VARIANCE**	**UP TO 5 MM**

**Source:** Prepared by the authors.

**Figure 8:**
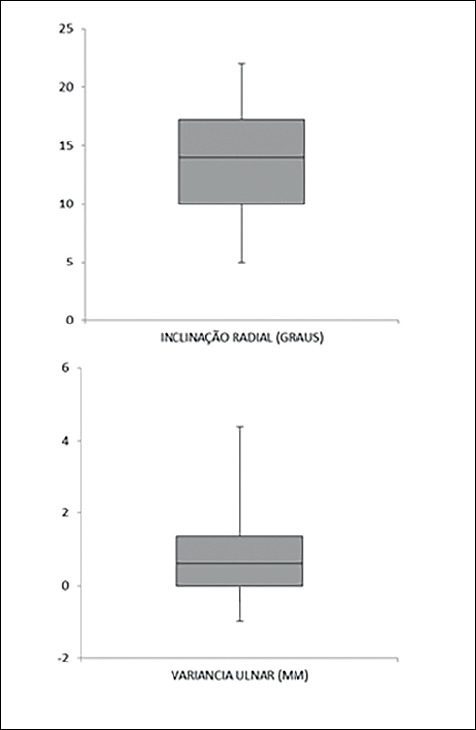
Box plot representations of obtained results for radial inclination and ulnar variance after treatment with spanning plates in the patients observed. Midlines represent medians, boxes represent standard errors, and vertical lines represent standard deviations.

## DISCUSSION

We present a solution for a problem (complex unstable fractures of the distal radius) many procedures and techniques tried to solve. Currently, there are blocked plates, which show good results, but are not available in all departments. Maintaining stability is as important as reducing the fracture, since extreme comminution leaves them prone to collapse and malunion can lead to numerous complications, mainly post-traumatic osteoarthritis. ^(^ ^7^ ^),(^ ^15^

Spanning plates, in addition to reducing the fracture, keep it stable, maintaining radius length and allowing the articular surface to consolidate without collapsing. A disadvantage is the plate needs to be removed after a new procedure, which can cause material fatigue and breakage if the plate is maintained for more than six months. ^(^ ^7^ ^),(^ ^8^

Compared to external fixators, spanning plates do not keep open wounds on the skin or a bulky external device, reducing complications and facilitating patient management both in and outside the hospital. ^(^ ^8^ Furthermore, Wolf et al. ^(^ ^16^, via a biomechanical test, showed greater rigidity and stability when using spanning plates, compared to external fixators, as long as three proximal screws and three distal screws were used.

Two recent anatomical studies examined the risk of iatrogenic damage to the radial sensory nerve when using a spanning plate. Lewis et al. ^(^ ^17^, after dissecting six cadavers, totaling 12 wrists, created unstable distal radius fractures, which were treated with a dorsal spanning plate in the second or third metacarpals. Compression of the radial sensory was not identified in none of them, but nerve branches were visualized when accessing fixation in the second metacarpal. Similarly, Dahl et al. ^(^ ^18^ ^)^ dissected 12 frozen cadavers and found branches of the radial sensory nerve were at risk when the second metacarpal was exposed and came into contact with the plate, which did not occur when the third ray was fixated.

Lauder et al. ^(^ ^19^ ^)^ reported almost total recovery of grip and extension strength after removing the spanning plate, especially in patients with wrist fractures on the dominant side.

The present study showed fixation of complex distal radius fractures using spanning plates reached solid results in terms of radiographic parameters. A satisfactory reduction was obtained in 11 of 14 patients, representing 78.5% of the total sample. Some study limitations can be indicated, such as small sample size, lack of direct comparison with another fixation method, and lack of patients’ functional evaluation.

## CONCLUSION

Distal radius fractures are a relevant injury in our daily lives, and their incidence increases every year. Overall, literature shows good functional results and reduced complication rates with the studied when compared to others, such as the external fixator.

Fixation using dorsal distraction plates is an excellent alternative when managing complex distal radius fractures in departments where blocked plates are unavailable. It is an effective and low-cost method, showing satisfactory results, especially regarding postoperative radiographic parameters. Therefore, it is a useful technique and should be present in the arsenal of hand surgeons and orthopedists.
